# A cross-sectional study of Australian teachers’ health: are work-related factors associated with lifestyle behaviours?

**DOI:** 10.1093/heapro/daad192

**Published:** 2024-01-10

**Authors:** Lucy Corbett, Philayrath Phongsavan, Anthony D Okely, Louisa R Peralta, Adrian Bauman

**Affiliations:** Sydney School of Public Health, The Charles Perkins Centre, The University of Sydney, John Hopkins Drive, Camperdown, 2050, Australia; Sydney School of Public Health, The Charles Perkins Centre, The University of Sydney, John Hopkins Drive, Camperdown, 2050, Australia; Early Start, Faculty of Social Sciences, University of Wollongong, Northfields Ave, Wollongong, New South Wales, 2522, Australia; Illawarra Health and Medical Research Institute, Northfields Ave, Wollongong, New South Wales, 2522, Australia; Sydney School of Education and Social Work, The University of Sydney, Manning Rd, Camperdown, 2050, Australia; Sydney School of Public Health, The Charles Perkins Centre, The University of Sydney, John Hopkins Drive, Camperdown, 2050, Australia

**Keywords:** school teachers, health behaviour, lifestyle, exercise, occupational diseases

## Abstract

Teacher’s lifestyle behaviours are important because they lead to positive health outcomes for teachers themselves and because teachers model behaviour to their students. This cross-sectional study examined the lifestyle behaviours of a large sample of teachers in New South Wales (NSW), Australia and assessed the association between work-related factors and lifestyle behaviours. From February to October 2021, data were collected on the lifestyle behaviours, work-related factors and socio-demographics of primary and secondary school teachers in NSW, via an online survey. Associations between individual work-related factors and lifestyle behaviours were modelled using logistic regression and adjusted for sex, age, number of children and geographic location. Most of our survey sample (*n* = 1136) were women (75%) and 53% were reported as having overweight or obesity. Only 23% of teachers met the recommended physical activity guidelines, 39% met fruit intake guidelines, 9% met vegetable intake guidelines and 58% met healthy sleep guidelines. Most teachers (78%) met the recommendation of sugar-sweetened beverage consumption, 89% were not current smokers, but only 46% met the recommended alcohol consumption guidelines. Hours worked, teaching load, school sector and teacher role were associated with one or more lifestyle behaviours after adjusting for the demographic variables. This study highlights the need for additional support to improve the health-related behaviours of teachers in NSW. Policymakers should recognize the negative impact of high workloads on teachers’ health-related behaviours, increasing their risk of chronic disease.

**Contribution to Health Promotion**

- Teachers’ lifestyle behaviours are a crucial factor in reducing morbidity, enhancing wellbeing and positively shaping the health of the next generation through role modelling.
- Only 23% of teachers met physical activity, 9% met vegetable, 39% met fruit and 58% met healthy sleep guidelines indicating a pressing need for greater support in fostering positive lifestyle behaviours among teachers in NSW.
- Workload, school sector and role significantly affect teachers’ ability to adopt healthy habits.
- To truly prioritize teachers’ health, policymakers must institute substantial and sustainable changes in the education system, starting with reducing heavy workload burdens.

## INTRODUCTION

The relationship between mortality, chronic disease and lifestyle behaviours such as physical activity, diet, smoking, alcohol consumption and sleep have been extensively researched (Cappuccio *et al*., 2011; Shield *et al.*, 2014; Lacombe *et al.*, 2019). For example, evidence shows physical activity influences a person’s risk of cardiovascular disease (Wahid *et al.,* 2016) and cancer, including breast (Monninkhof *et al.*, 2007), colon (Boyle *et al*., 2012) and renal (Behrens and Leitzmann, 2013) cancers. Participating in multiple unhealthy behaviours increases the risk of all-cause mortality (Ding *et al*., 2015). In contrast, evidence shows the positive impact a healthy lifestyle can have on reducing the risk of chronic illnesses (Bauer *et al.*, 2014).

Teachers’ health-related behaviours are important because they lead to positive health outcomes for teachers themselves and because teachers model health behaviours to their students (Bevelander *et al*., 2013; Findholt *et al.,* 2016; Hamilton *et al.*, 2021). Teachers’ own health behaviours may influence their confidence in delivering health education programs in schools (Kennedy *et al*., 2019; Herlitz *et al*., 2020). Despite this, few studies have explored teachers’ health behaviours and lifestyles. Findings from the limited studies exploring teachers’ lifestyle behaviour are varied. Several international studies reported better lower levels of tobacco smoking (Ohida *et al.,* 2000; Gilbert *et al*., 2015; Riechmann-Wolf *et al.,* 2021; Temam *et al*., 2022) and cannabis use (Gilbert *et al.*, 2015; Temam *et al.*, 2022) compared with the general population. However, physical activity levels have been reported either as low (Dias *et al.*, 2017), similar (Temam *et al.*, 2022) or higher (Riechmann-Wolf *et al.*, 2021) compared with the general population or other occupational groups. Alcohol consumption by teachers has been reported as lower (Temam *et al.*, 2022), no different (Gilbert *et al.*, 2015) and higher (Riechmann-Wolf *et al*., 2021) than non-teachers. An Israeli study found teachers to have unfavourable health behaviours and high levels of obesity (Wilf-Miron *et al*., 2022). There are few large population surveys in this area but the prevalence of obesity amongst German kindergarten teachers has been reported as higher than the general population (Hoffmann *et al.,* 2013), whereas a nationwide French survey found teachers were less likely to be overweight or obese, compared with other occupations (Gilbert *et al*., 2015; Temam *et al*., 2022).

Teaching is a demanding profession and work-related factors such as workload, employment contract and other working conditions, which may vary by local country, could potentially contribute to these conflicting findings. Previous research has shown that work-related factors such as working part-time may influence adult workers’ participation in healthy lifestyle behaviours (Mein *et al*., 2005; Kirk and Rhodes, 2011). However, very few studies have considered the impacts of work-related factors on teachers’ lifestyle behaviours (Dias *et al*., 2017; Temam *et al.*, 2022). One study found the length of employment contract was associated with teachers’ physical activity levels (Dias *et al.*, 2017). A study of French teachers (Temam *et al*., 2022) found secondary school teachers were more likely to be at-risk drinkers than their primary school colleagues. Further research into teacher lifestyles and the impact work-related factors have on these lifestyles is necessary to advocate for systemic changes that will have a positive impact on teachers’ health and determine priority areas for intervention.

This study examined the lifestyle behaviours of teachers in New South Wales (NSW), Australia, and evaluated the associations between work-related factors and their lifestyle behaviour. We hypothesized that work-related factors such as teaching load may be associated with lifestyle behaviours. At the time of this study, the coronavirus disease of 2019 (COVID-19) was an additional challenge for teachers. COVID-19 had a negative impact on lifestyle behaviours (Stockwell *et al.*, 2021) and reduced levels of physical activity among the adult working population (Ráthonyi *et al*., 2021). Due to the substantial workplace differences between teaching and other professional groups, it is important to capture teachers’ perceived risk of COVID-19 and its impact on engagement in lifestyle behaviours. Therefore, a secondary aim of this study was to determine whether the perceived risk of COVID-19 was associated with teachers’ lifestyle behaviours.

## METHODS

The STROBE statement (von Elm *et al.*, 2007) has been used in the reporting of this study.

### Research design

This cross-sectional study used an online survey to collect information on lifestyle behaviours, mental well-being, work-related factors and socio-demographics from primary and secondary school teachers in NSW, Australia. A stratified single-stage cluster sampling design was used to generate a sample of schools from which to recruit teachers. Schools were sampled with probabilities proportional to the number of teaching staff from the list of all schools in NSW. The sample of schools to be sampled was estimated to be 220 based on an expected proportion of = 0.5, desired precision = 0.05, α = 0.05, expected intraclass correlation = −0.2, number of teachers in NSW = 84,500 and average number of teachers per school = 29. Emails were sent to principals of 500 randomly selected schools providing information about the study and inviting them to forward the survey link to teaching staff at their school. However, due to the difficulties arising from COVID-19 and the influence the pandemic had on schools, the recruitment process had to forgo random sampling and instead rely on convenience sampling. The revised sample size calculation used an expected proportion = 0.5, desired precision = 0.05 and α = 0.05 and provided a target sample size of 385 teachers. The survey was also promoted through internal communications from various professional teaching organizations to their members (e.g. Professional Teachers’ Council NSW). Targeted Facebook advertising was also used to reach teachers who were over 20 years old. To prevent online bots from accessing the survey, a reCAPTCHA test was used. The survey opened on 4 February 2021 and closed on 12 October 2021. To be eligible to complete this study, participants had to be currently employed as a teacher at a school in NSW.

The research was approved by the Human Research Ethics Committee, The University of Sydney (Protocol No. 202/325).

### Measures

#### Lifestyle behaviours

Physical activity in the previous week was measured using a validated single-item question (Milton *et al.,* 2011). Meeting physical activity guidelines was classified as participating in at least 30 min of physical activity ≥ 5 days/week which is in line with interpretations of the Australian physical activity guidelines for adults (Australian Department of Health and Aged Care, 2021).

Daily fruit, vegetable and sugar-sweetened beverage (SSB) intake was reported using questions from the NSW Population Health Survey, 2019 (Centre for Epidemiology and Evidence, 2019). The validity of these questions has been shown to be good when compared with 24-h recalls (Barr *et al*., 2008). In line with the Australian dietary guidelines, consuming ≥ 2 servings of fruit/day and ≥ 5 servings of vegetables per day were classified as meeting guidelines (National Health and Medical Research Council, 2013). SSB intake was categorized into daily or less than daily consumption.

Cigarette and e-cigarette smoking status was measured using two previously validated (Barr *et al*., 2008) questions from the NSW Population Health Survey (Centre for Epidemiology and Evidence, 2019). Those who smoked either cigarettes or e-cigarettes daily or occasionally were classified as current smokers.

Alcohol consumption was measured using validated (Barr *et al.*, 2008) questions from the NSW Population Health Survey. As reflected by the 2020 national guidelines (National Health and Medical Research Council, 2020) those who consumed more than 10 standard drinks in a week or more than four standard drinks on one occasion were classified as not meeting guidelines.

Self-reported height (cm) and weight (kg) were used to calculate body mass index (BMI, kg/m^2^). BMI was classified into underweight/healthy weight (BMI < 25 kg/m^2^) and overweight/obesity (BMI ≥ 25 kg/m^2^).

Sleep duration was assessed by asking participants about what time they usually go to bed and what time they usually wake (Adams *et al.*, 2017). The National Sleep Foundation’s guidelines, sleeping for 7–9 h/night on average (Hirshkowitz *et al.*, 2015) was classified as meeting sleep guidelines for adults.

#### Work-related factors

Participants were asked several questions about their employment including: the year that they were first employed as a teacher (teaching experience), what educational sector (independent, government or Catholic) their current school was a part of, the type of school they taught at (primary or secondary), whether they had a full-time or part-time load (teaching load), whether their employment contract was permanent, fixed-term or casual (contract type), whether they taught primary or secondary school students (teacher type) and whether they had a leadership position (e.g., head of department, year co-ordinator, deputy principal, principal) at school (teacher role). A single-item question adapted from the Australian Teacher Workforce Data Survey 2020 (Australian Institute for Teaching and School Leadership, 2021) was used to measure hours worked in an average week.

The perceived risk of COVID-19 was measured using an adapted question (Gentili *et al.*, 2020). Participants were asked to rate their perceived risk of COVID-19 as no, low, moderate or high risk (COVID risk).

#### Demographics

Demographic information including sex, age, number of children and geographical location was collected. The complete questionnaire is found in Supplementary Material S1.

### Statistical analysis

A healthy lifestyle index was created by combining physical activity, fruit consumption, vegetable consumption, SSB consumption, smoking behaviour and sleep lifestyle behaviours in a single variable. This method was adapted from methods developed by Ding and colleagues (Ding *et al.*, 2014, 2015). Alcohol consumption was excluded due to the higher number of people who preferred not to answer questions pertaining to alcohol consumption, however, the sensitivity analysis can be found in Supplementary Material S2. The healthy behaviours (e.g., meeting guidelines, not-smoking) were coded 1 so that when the lifestyle behaviours were combined, a higher score indicated that the individual had healthier behaviour. Individuals with scores of 3 or less were classified as ‘unhealthy’ while those with scores of 4 or higher were deemed ‘healthy’.

Descriptive statistics were provided for the sample, and the prevalence of each lifestyle behaviour was reported by demographic characteristics and work-related factors. Associations between individual work-related factors and lifestyle behaviours were modelled using logistic regression and adjusted for sex, age, having children and geographical location since these demographics are known to influence multiple lifestyle behaviours (Kendig *et al.,* 2007; Matthews *et al*., 2017). Since the missing data for most variables were < 10%, missing data were excluded during analysis.

All analysis was conducted using R version 4.0.3 with epiR (version 1.0.15), car (version 3.0.9) and jtools (version 2.1.0) packages.

## RESULTS

### Demographic characteristics

In our survey sample (*N* = 1136), most were women (75%) and the BMI was 27.7 ± 6.1 kg/m^2^. Thirty percent (30%) of the teachers had a BMI classified as underweight or healthy weight. Fifty-five percent (55%) of the teachers taught at schools in suburban areas and 55% perceived the risk from COVID-19 to be moderate or high at the time of completing the survey (Table 1).

**Table 1:** Proportion of teachers by demographic and work-related factors participating in certain lifestyle behaviours, *N* = 1136

	*N* (%)	Meeting PA guidelines (%)	Meeting fruit guidelines (%)	Meeting vegetable guidelines (%)	Does not drink SSB daily (%)	Non-smoker (cigarette or e-cigarette) (%)	Meeting alcohol guidelines (%)	Meeting sleep guidelines (%)	Classified as healthy on lifestyle index**
**Sex** Female Male Missing	857 (75) 179 (16) 100 (9)	21.1 30.7	40.8 40.3	10.5 6.7	79.9 76.0	92.7 91.1	62.4 44.2	64.1 55.9	33.7 27.4
**Age group** <35 years 35–49 years >50 years Missing	265 (23) 403 (35) 363 (32) 105 (9)	20.8 22.6 25.1	34.3 37.5 47.1	6.0 9.2 13.3	72.1 77.2 84.6	92.1 91.3 93.7	57.3 59.6 60.4	65.0 60.7 63.0	25.1 31.3 39.1
**Geographical location** Urban Suburban Rural/remote Missing	199 (18) 624 (55) 313 (28) 0 (0)	31.1 20.9 22.0	40.2 40.4 40.7	8.8 9.2 11.3	76.8 78.3 81.0	91.8 92.6 92.3	56.8 62.0 56.5	61.0 63.9 59.6	34.2 32.3 32.8
**Have children** No Yes Missing	547 (48) 482 (42) 107 (9)	24.5 21.4	41.7 38.4	10.2 9.8	80.3 75.9	93.4 91.7	57.5 61.6	62.9 62.1	35.48 29.9
**BMI** Underweight/healthy weight Overweight/obesity Missing	355 (31) 597 (53) 184 (16)	29.0 18.1	43.9 38.4	11.3 9.1	85.4 75.9	90.7 93.5	67.9 53.6	68.3 59.6	39.9 27.6
**Perceived risk of COVID-19** No/low risk Moderate risk High risk NA	327 (29) 427 (38) 193 (17) 184 (17)	23.1 18.6 34.7	38.1 43.5 41.4	9.6 12.0 7.3	76.2 81.6 77.0	93.5 92.1 91.1	60.4 60.8 53.3	58.3 65.0 57.9	29.6 36.1 31.7
**Hours worked/week** <40 h/week 40–49 h/week 50–59 h/week 60 + h/week Missing	170 (15) 230 (20) 423 (37) 273 (24) 40 (4)	29.9 24.6 19.7 21.3	47.6 38.1 42.9 32.3	11.5 8.5 9.4 9.9	85.5 83.0 76.3 73.8	91.0 92.4 93.0 92.4	68.5 56.7 53.8 64.7	53.9 72.6 63.0 51.8	40.5 36.1 32.8 23.2
**Teaching experience** <5 years 6–15 years 16 + years Missing	164 (14) 340 (30) 632 (56) 0 (0)	22.0 20.3 24.6	34.4 39.2 42.7	8.3 8.1 11.0	72.6 76.5 81.5	87.3 93.4 93.1	57.6 58.3 60.7	61.3 62.9 62.1	25.2 31.3 35.6
**Teaching load** Full time Part time Missing	898 (79) 238 (21) (0)	22.7 24.1	37.2 52.3	8.9 12.8	78.1 91.3	91.1 97.0	57.3 67.9	61.1 66.2	29.9 43.0
**Contract type** Permanent Fixed term/casual Missing	839 (74) 297 (26) 0 (0)	21.9 25.9	40.2 41.0	9.3 11.0	79.8 75.9	92.2 92.8	59.5 59.6	62.13 32.5	32.5 33.3
**School sector** Independent Government Catholic Missing	183 (16) 726 (64) 227 (20) 0 (0)	37.9 20.1 19.7	39.4 40.5 41.1	10.6 8.8 11.9	82.2 77.2 80.8	90.0 92.4 94.1	66.4 58.0 58.9	62.4 62.0 63.0	64.6 68.1 66.8
**Teacher role** Teacher Leadership position (HOD, deputy, principal) Missing	840 (74) 296 (26) 0 (0)	23.7 20.9	42.7 34.0	10.3 8.0	78.4 79.9	92.7 91.3	61.1 55.1	63.1 59.6	31.0 29.1
**Teacher type** Primary Secondary Missing	573 (42) 563 (50) 94 (8)	21.6 24.4	40.9 39.9	10.3 9.1	76.3 81.2	7.8 7.5	62.4 56.8	61.2 63.2	33.7 31.8

Legend: ** score 4 + healthy lifestyle habits.

### Lifestyle behaviours

Only 23% of teachers surveyed met the recommended physical activity guidelines. Thirty-nine percent (39%) met fruit intake guidelines, 9% met vegetable intake guidelines and 58% met healthy sleep guidelines. Most teachers (78%) did not drink SSBs daily, 89% were not current smokers, but only 46% met the recommended alcohol guidelines. Table 1 shows that a higher proportion of females met lifestyle behaviour guidelines compared with males except for physical activity (21% females vs. 31% males). Additionally, a higher proportion of teachers in older age ranges tended to report a healthy lifestyle behaviour score, compared with those in the younger age ranges (39% vs. 25%, respectively).

### Association between work-related factors and lifestyle behaviour

Table 2 shows that the number of hours worked per week was significantly associated with meeting fruit intake (*p* = 0.015, χ^2^_(3)_ = 10.52), SSB consumption (*p* = 0.015, χ^2^_(3)_ = 10.50) and sleep recommendations (*p* < 0.001, χ^2^_(3)_ = 17.83). Teachers who worked 40–49, 50–59 and 60 + h/week all had lower odds of meeting the recommended fruit intake guidelines (OR_(40–49 hours/week)_ = −0.70; OR_(50–59 hours/week)_ = −0.89; OR_(60+ hours/week)_ = −0.54) and lower odds of having healthy SSB consumption (OR_(40–49 hours/week)_ = −0.84; OR_(50–59 hours/week)_ = −0.50; OR_(60+ hours/week)_ = −0.50) compared with teachers who worked less than 40 h/week. Teachers who worked over 60 h/week had lower odds (OR = 0.72; 95%CI: 0.46–1.13) of meeting sleep guidelines compared with those who worked less than 40 h/week. Teaching part-time was associated with meeting fruit intake guidelines (*p* < 0.001, χ^2^_(1)_ = 11.45) and currently not smoking (*p* = 0.003, χ^2^_(1)_ = 8.60). While part-time teachers tended to have better lifestyle behaviours than their full-time colleagues, no other significant associations between teaching load and lifestyle behaviours were found.

**Table 2:** Odds ratios (ORs) and 95% confidence intervals (95%CI) of meeting recommended guidelines for lifestyle behaviours in relation to work-related factors, adjusting for sex, age, BMI, geography and having children

Work-related factors	Meeting PA guidelines Odds ratio (95%CI)	Meeting fruit guidelines Odds ratio (95%CI)	Meeting vegetable guidelines Odds ratio (95%CI)	Does not drink SSB daily Odds ratio (95%CI)	Non-smoker (cigarette or e-cigarette) Odds ratio (95%CI)	Meeting alcohol guidelines Odds ratio (95%CI)	Meeting sleep guidelines Odds ratio (95%CI)
**Hours worked/week** <40 h (ref) 40–49 h 50–59 hours 60 + hours	1.0 0.73 (0.44–1.23) 0.67 (0.42–1.07) 0.80 (0.49–1.33)	* 1.0 0.70 (0.45–1.10) 0.89 (0.60–1.34) 0.54 (0.34–0.84)	1.0 0.88 (0.43–1.82) 0.88 (0.43–1.82) 0.91 (0.47–1.74)	* 1.0 0.84 (0.45–1.55) 0.50 (0.29–0.87) 0.50 (0.28–0.91)	1.0 1.35 (0.62–2.98) 1.38 (0.68–2.80) 1.13 (0.53–2.44)	1.0 0.72 (0.43–1.21) 0.65 (0.41–1.04) 1.01 (0.60–1.71)	*** 1.0 1.76 (1.09–2.85) 1.05 (0.69–1.59) 0.72 (0.46–1.13)
**Teaching experience** <5 years (ref) 6–15 years 16 + years	1.0 0.94 (0.56–1.60) 1.16 (0.63–2.13)	1.0 1.10 (0.70–1.71) 1.14 (0.68–1.90)	1.0 0.64 (0.30–1.35) 0.56 (0.24–1.28)	1.0 1.17 (0.71–1.91) 1.46 (0.81–2.62)	1.0 1.79 (0.88–3.67) 2.27 (0.99–5.18)	1.0 0.90 (0.55–1.47) 1.03 (0.58–1.84)	1.0 1.10 (0.71–1.71) 1.26 (0.75–2.10)
**Teaching load** Full time (ref) Part time	1.0 0.96 (0.65–1.41)	*** 1.0 1.74 (1.26–2.39)	1.0 1.44 (0.88–2.35)	1.0 1.16 (0.77–1.74)	** 1.0 2.91 (1.30–6.50)	1.0 1.20 (0.83–1.74)	1.0 1.13 (0.81–1.58)
**Contract type** Permanent (ref) Fixed term/casual	1.0 1.30 (0.92–1.83)	1.0 1.08 (0.80–1.46)	1.0 1.51 (0.94–2.4)	1.0 0.87 (0.60–1.25)	1.0 1.16 (0.66–2.05)	1.0 1.01 (0.72–1.42)	1.0 1.03 (0.76–1.40)
**School Sector** Independent (ref) Government Catholic	*** 1.0 0.49 (0.33–0.72) 0.39 (0.24–0.66)	1.0 1.15 (0.79–1.66) 1.16 (0.75–1.81)	1.0 0.79 (0.44–1.41) 0.87 (0.43–1.75)	1.0 0.77 (0.48–1.23) 0.94 (0.54–1.66)	1.0 1.57 (0.86–2.87) 1.94 (0.87–4.31)	1.0 0.76 (0.49–1.16) 0.83 (0.50–1.36)	1.0 1.00 (0.69–1.45) 1.27 (0.80–2.00)
**Teacher role** Teacher (ref) Leadership position	1.0 0.80 (0.55–1.16)	* 1.0 0.69 (0.50–0.94)	1.0 0.59 (0.33–1.03)	1.0 0.99 (0.68–1.45)	1.0 0.91 (0.52–1.60)I	1.0 0.84 (0.60–1.19)	1.0 0.92 (0.67–1.25)
**Teacher type** Primary Secondary	1.0 0.89 (0.64–1.23)	1.0 0.93 (0.71–1.22)	1.0 0.84 (0.53–1.32)	1.0 1.32 (0.94–1.85)	1.0 1.02 (0.62–1.69)	1.0 0.94 (0.69–1.28)	1.0 1.17 (0.88–1.54)
**Perceived risk of COVID–19** No/low risk (ref) Moderate risk High risk	*** 1.0 0.75 (0.51–1.12) 1.82 (1.16–2.85)	1.0 1.18 (0.86–1.63) 1.11 (0.74–1.67)	1.0 1.39 (0.83–2.34) 0.70 (0.32–1.51)	1.0 1.25 (0.84–1.86) 1.04 (0.64–1.69)	1.0 0.58 (0.31–1.11) 0.48 (0.23–1.02)	1.0 1.01 (0.71–1.44) 0.72 (0.45–1.15)	1.0 1.43 (1.03–1.98) 1.05 (0.70–1.57)

* = *p* < 0.05, ** = *p* < 0.01, *** = *p* < 0.001.

Teachers at government (OR = 0.49; 95%CI: 0.33–0.72) and Catholic schools (OR = 0.39; 95%CI: 0.24–0.66) had lower odds of meeting physical activity guidelines (*p* < 0.001, χ^2^_(2)_ = 15.68), compared with teachers from independent schools. Teachers in a leadership position had lower odds of meeting fruit guidelines (OR = 0.69; 95%CI: 0.50–0.94; *p* = 0.018; χ^2^_(1)_ = 5.56), compared with teachers not in a leadership position.

The perceived risk of COVID-19 was associated with meeting physical activity guidelines (*p* < 0.001, χ^2^_(2)_ = 15.20). Teachers who perceived the risk of COVID-19 to be high had higher odds of meeting physical activity guidelines (OR = 1.82; 95%CI: 1.16–2.85), compared with those who perceived the risk as low. No significant associations were found between teacher experience, teaching role, contract type or school type and any lifestyle behaviours.

### Association between work-related factors and healthy lifestyle index

**Fig. 1:**
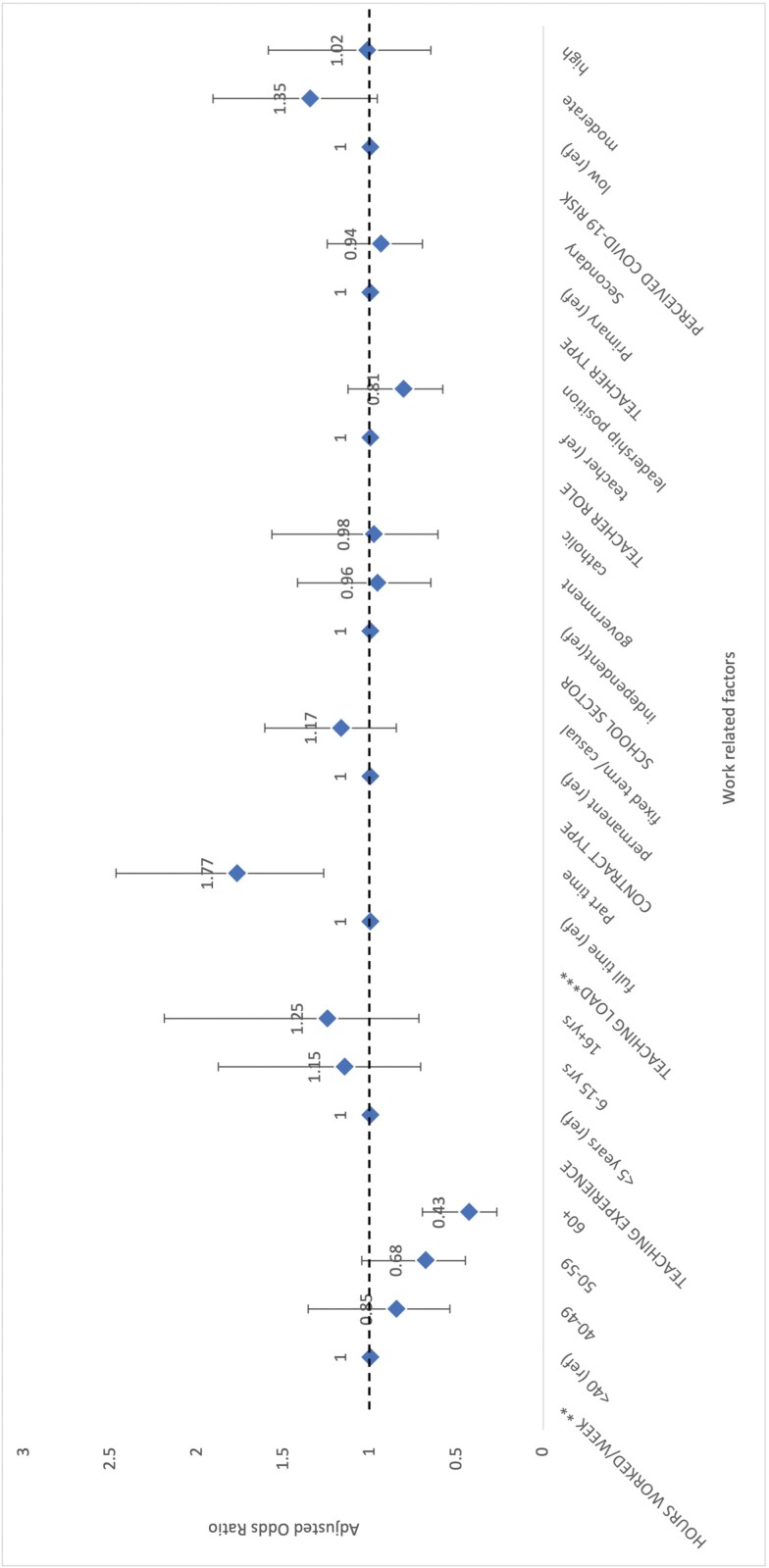
Odds of being classified as ‘healthy’ on lifestyle index based on various work-related factors after adjusting for sex, age, BMI, geography and having children. Cut point of 4 + healthy lifestyle behaviours were used to classify teachers as healthy. *p < 0.05, **p < 0.01, ***p < 0.001.

The associations between the various work-related factors and being classified as ‘healthy’ on the lifestyle index after adjusting for sex, age, BMI, geography and having children are shown in Figure 1. The number of hours worked per week (*p* = 0.002, χ^2^_(3)_ = 14.59) and teaching load were both associated with the healthy lifestyle index. Teachers who worked more than 40 h/week had lower odds (OR_(40–49 hours/week)_ = −0.85; OR_(50–59 hours/week)_ = −0.68; OR_(60+ hours/week)_ = −0.43) of having a healthy lifestyle index, compared with teachers who worked less. Part-time teachers had higher odds (OR = 1.77; 95%CI: 1.27–2.47; *p* < 0.001, χ^2^_(1)_ = 11.29) of being classified as healthy. While teaching experience was not significantly associated with the healthy lifestyle index, a trend shows teachers with 6–15 years and over 16 years of teaching experience had higher odds of being classified as healthy, compared with teachers who had less than 5 years of experience. Teachers with a leadership role had, on average, lower odds of being classified as healthy, although this trend was not significant. There was no significant association between school sector, teacher type or perceived COVID-19 risk and lifestyle index.

## DISCUSSION

This study identifies the importance of teachers’ physical activity levels and lifestyle behaviours. On average one in four teachers in this survey met the physical activity guidelines compared with an estimated 62% of the general adult population in NSW (Centre for Epidemiology and Evidence, 2019). The disparity may partially be accounted for by the impact of COVID-19, which has been reported worldwide as reducing physical activity levels (Stockwell *et al*., 2021), as the data from the NSW population estimate were collected prior to the COVID-19 pandemic. The prevalence of overweight and obesity was similar among teachers in our study (53%), compared with the adult population in NSW (57%) (Centre for Epidemiology and Evidence, 2019). While the proportion of teachers who met fruit (39%) and vegetable (9%) intake guidelines were low, these figures are comparable with the general adult population in NSW with 40% meeting fruit and 6% meeting vegetable intake guidelines (Centre for Epidemiology and Evidence, 2019). Compared with the general Australian population, a lower proportion of teachers in our sample met healthy alcohol consumption guidelines (46% vs. 74%) (Australian Bureau of Statistics, 2022) and more teachers consumed SSB daily (24% vs. 9%) (Australian Bureau of Statistics, 2018).

A previous Australian study measured alcohol consumption among teachers, however, the sample size was small with 166 participants (Stapleton *et al*., 2020). Despite using convenience sampling techniques, our large sample of teachers is reflective of the broader population of teachers in NSW. Our sample is made up of 75% female teachers, 32% are over 50 years, 28% teach at schools in rural or remote areas, 64% taught at government schools and 74% have permanent contracts. This is comparable with the general NSW teaching population which is made up of 78% female teachers, 37% over 50 years, 32% teach in rural or remote areas, 63% teach at government schools and 69% have permanent contracts (Australian Institute for Teaching and School Leadership, 2021). Whilst convenience sampling reduces the generalisability of results, the similarities between characteristics in our sample and the population of NSW teachers indicate that our sample is typical of teachers in NSW.

This study contributes to the relatively new line of research assessing the impact of work-related factors on teachers’ lifestyle behaviour. A previous study found Brazilian teachers on temporary contracts had lower levels of physical activity than their permanent colleagues (Dias *et al*., 2017) and a French study found secondary school teachers were more likely to have at-risk alcohol consumption than primary teachers (Temam *et al*., 2022). Whilst this study found associations between several work-related factors and various lifestyle behaviours, no association between contract type or teacher type and any lifestyle behaviours was found. Just as a teacher’s workload varies by country (OECD, 2021), our finding indicates that work-related factors impacting teachers’ health also vary by local contexts. Our study found that hours worked per week and teaching load were significantly associated with healthy lifestyle behaviours (Figure 1), which demonstrates the negative impact that limited leisure time may have on teachers’ health. A lack of time has been cited as a barrier to healthy lifestyle behaviour amongst various populations (Moreno and Johnston, 2014; Burgess *et al.*, 2017) and qualitative research has described the frustration teachers feel at not having time to be physically active or eat healthily (McCallum *et al*., 2010).

Currently, NSW is facing a teacher shortage crisis (Rose and McGowan, 2022), there is an increasing awareness of issues of early career attrition and teacher retention (Rajendran *et al.*, 2020), and the fact that large workloads are repeatedly cited as a source of teacher stress and attrition (Heffernan *et al.*, 2019). Our findings suggest that a high workload may also negatively influence teachers’ health-related behaviours, potentially reducing teachers’ confidence when implementing health promotion programs (Kennedy *et al.,* 2019; Herlitz *et al.*, 2020) and contributing to chronic disease in the future. Policymakers should consider implementing measures to alleviate the workload burden on teachers. Several strategies merit consideration to ease teachers’ workload burdens, including finding ways other school staff, can take on non-teaching work such as extra-curricular activities or duties, streamlining or automating administrative tasks, and providing high-quality resources for curriculum planning (Hunter *et al*., 2022). Further research is needed to identify which specific tasks, if modified or alleviated, would yield the most substantial benefits for teachers (Thompson *et al*., 2023). Such insights would enable more targeted interventions and policies to improve teachers’ overall work experience.

COVID-19 was an additional complexity for teachers to navigate at the time of this study. Over half of our sample perceived the risk of COVID-19 to be moderate or high. Teachers who perceived COVID-19 to be a high risk were 82% more likely to meet physical activity guidelines, compared with those who perceived it to be low risk after adjusting for geographical location, children, age, sex and BMI. Interestingly, no association was found between perceived COVID-19 risk and alcohol consumption among teachers, despite higher levels of alcohol consumption reported among Australian adults during the pandemic (Callinan *et al*., 2021). Overall, the perceived risk of COVID-19 was not associated with other lifestyle behaviours or being classified as healthy.

### Limitations of this study

This was a cross-sectional study which was appropriate for providing a snapshot of the lifestyle behaviours among NSW teachers. Cross-sectional studies measure both explanatory and outcome variables simultaneously, and hence do not provide evidence of a causal relationship; future studies need longitudinal data to assess whether work-related factors cause better or worse lifestyle behaviours. However, these analyses do point to the need for lifestyle interventions in this large occupational group.

COVID-19 impacted the collection of data. Initially, a stratified single-stage cluster sampling design was planned to collect a representative sample of NSW teachers. However, due to the profound impact COVID-19 had on schools and the ability to conduct research within schools, a convenience sampling method was utilized. Whilst the researchers tried to reach teachers through different channels, convenience sampling may have introduced bias into the data (Etikan *et al*., 2016). Thus, care should be taken when interpreting these results. In Australia especially, future studies utilizing representative methods are needed to characterize the prevalence of lifestyle behaviours more accurately among teachers.

Self-report questionnaires are valuable tools and deemed appropriate for population surveillance and large cohort studies owing to their cost-effectiveness and ease of administration, however, there are a few weaknesses associated with their use, including susceptibility to social desirability bias and reduced validity (Krumpal 2011; Kelly *et al.,* 2016). However, in the context of large-scale investigations like this one, which has 1136 participants, the practicality of implementing objective measures is often limited and requires substantial funding. Whilst this study employed pre-validated measures to mitigate the shortcomings of self-report data, it is necessary to acknowledge and consider these limitations when interpreting our findings.

## CONCLUSION

Several work-related factors including hours worked, teaching load, school sector and teacher role were associated with one or more lifestyle behaviours after adjusting for demographic variables. After combining lifestyle behaviours into a single ‘healthy lifestyle index’, teachers’ who worked more hours per week and had a full-time teaching load were less likely to participate in multiple healthy lifestyle behaviours. Policymakers should be aware that a high workload may influence teachers’ health-related behaviour and chronic disease risk and take further action to reduce teachers’ workload. Future research aimed at promoting healthy lifestyles with teachers should consider work-related factors in the design of health promotion programs.

## Supplementary Material

daad192_suppl_Supplementary_Figures_S1

daad192_suppl_Supplementary_Figures_S2

## Data Availability

The data that support the findings of this study are available on reasonable request from the corresponding author. The data are not publicly available due to privacy or ethical restrictions.
