# Supplementary material for: A cross-sectional study of Australian teachers’ health: are work-related factors associated with lifestyle behaviours?
Source: Health Promot Int. 2024 Jan 10;39(1):daad192. doi: 10.1093/heapro/daad192 (PMC10781439; doi:10.1093/heapro/daad192)
Supplement: daad192_suppl_Supplementary_Figures_S1 [file daad192_suppl_supplementary_figures_s1.docx]

NSW Teachers’ Health Survey

I have read and understood the above statement and consent to participate in the NSW Teachers' Health Study.

Yes  (1)

No  (2)  (ineligible)

Are you currently employed as a teacher in NSW?

1. Yes
2. No (ineligible)

[PAGE BREAK]

First some information about your teaching

This **Teacher Survey** will ask you about your current experience.

Please remember to tell us about your 2020 teaching experience in the following questions.

SD1 In what year were you first employed as a teacher/educator?

SD2

Please use the drop down menu to select the approximate location of your school. You may use the map below to help you answer this question.

1. Capital Region
2. Central coast
3. Central West
4. Coffs Harbour – Grafton
5. Far West and Orana
6. Huner Valley exc Newcastle
7. Illawarra
8. Mid North Coast
9. Murray
10. New England and North West
11. Newcastle and Lake Macquarie
12. Richmond – Tweed
13. Riverina
14. Southern Highlands and Shoalhaven
15. Sydney – Baulkham Hills and Hawkesbury
16. Sydney – City and Inner South
17. Sydney – Eastern Suburbs
18. Sydney – Inner South West
19. Sydney – Inner West
20. Sydney – North Sydney and Hornsby
21. Sydney – Northern Beaches
22. Sydney – Outer South West
23. Sydney – Outer West and Blue Mountains
24. Sydney – Parramatta
25. Sydney – Ryde
26. Sydney – South West
27. Sydney – Sutherland


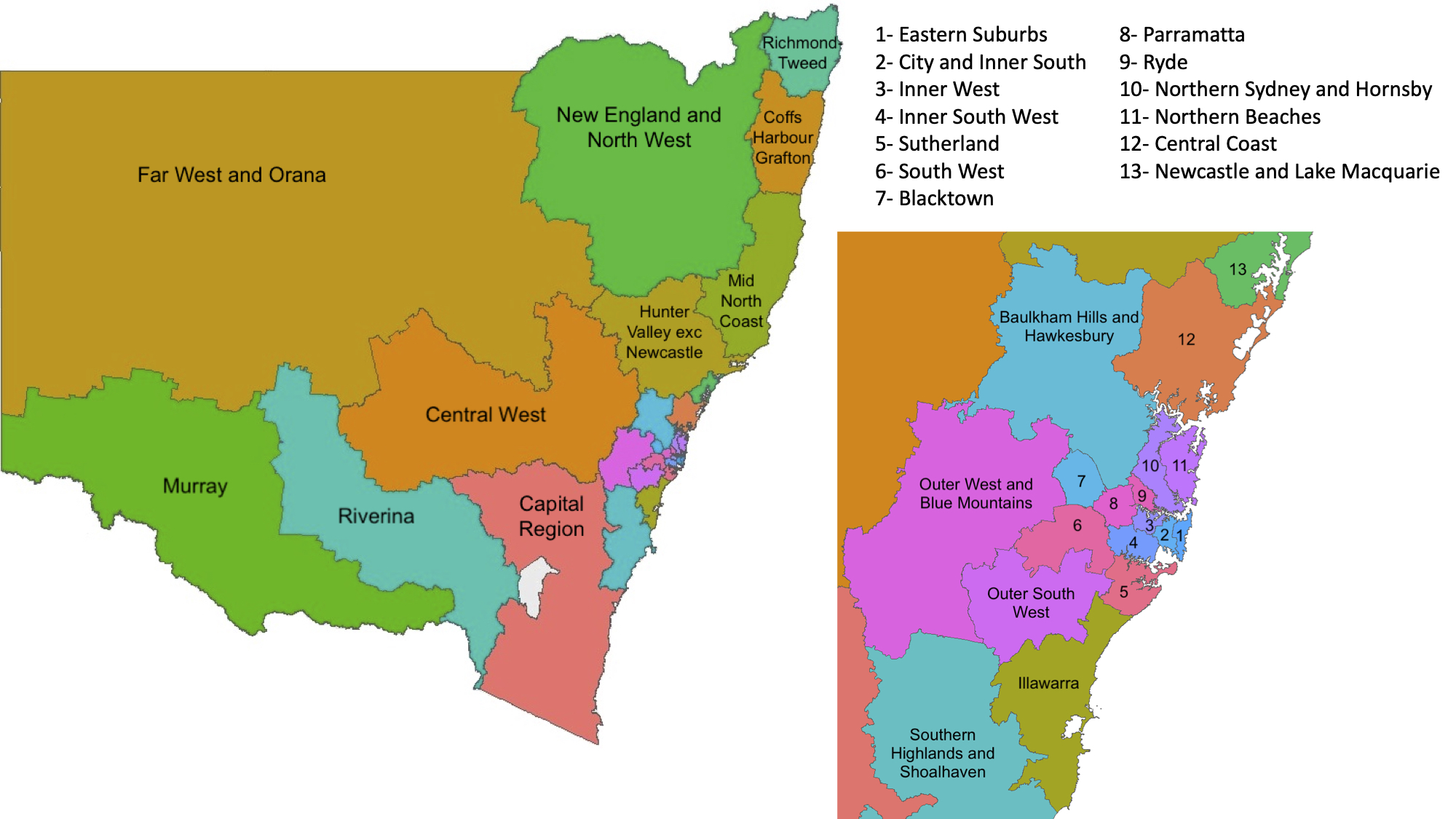


SD3 Please select the category that you think best describes the community where your school is

1. Urban
2. Suburban
3. Rural
4. Remote

SD4 What educational sector is your current school a part of?

1. Independent
2. Government
3. Catholic

SD5 Which of the following best describes your main role?

1. Teacher/ educator
2. Principal / Deputy principal
3. School leader other (e.g. Head of department, year coordinator)

SD6 Which of the following best describes your employment status?

1. Permanent employment (an on-going contract with no fixed end-point before the age of retirement)
2. Fixed-term contract for a period of more than 1 school year
3. Fixed-term contract for a period of 1 school year or less
4. Casual

SD7 What is your current employment status as a teacher, in terms of working hours?

1. Full-time (More than 90% of full-time hours)
2. Part-time (71-90% of full-time hours)
3. Part-time (50 – 70% of full-time hours)
4. Part-time (less than 50% of full-time hours)

SD8

Please tell us about your working hours during an average teaching week. An average teaching week is one that is not shortened by breaks, public holidays, sick leave etc.

Taking into account all the work done both inside and outside of work hours, during the average week, approximately how many hours (in total) did you work?

*This includes face-to-face teaching, planning lessons, marking, collaborating with other teachers, participating in staff meetings and other tasks related to your job that could take place during weekends, evenings or other 'out of school' hours.*

_______ hours

SD 9 Please select the stage(s) you are teaching

1. Stage 1 (Kindergarten – Year 2)
2. Stage 2 (Year 3 – year 4)
3. Stage 3 (Year 5 – Year 6)
4. Stage 4 (Year 7 – Year 8)
5. Stage 5 (Year 9 – Year 10)
6. Stage 6  (Year 11 – Year 12)
7. Not applicable

[PAGE BREAK]

The following questions will ask about your experience with recent natural disasters and disease outbreaks.

ND1 Were you affected by any of the following:

1. 2019/2020 bushfire (1)
2. 2019/ 2020 flooding (2)
3. COVID-19 (3)
4. Prefer not to say (4)

IF ND1=1

ND2 What was your perception of risk during the 2019/2020 bushfires?

1. No risk
2. Low risk
3. Moderate risk
4. High risk

ND3 What aspect of the 2019/2020 bushfires caused you the greatest concern? Select up to 3

1. Air quality
2. Damage to your property
3. Damage to the property of other family members
4. The need to evacuate
5. Risk of injury or illness to yourself
6. Risk of injury or illness to a family member
7. Economic impacts
8. Other concerns (please specify) ______

IF ND1=2

ND4 What was your perception of risk during the 2019/2020 floods?

1. No risk
2. Low risk
3. Moderate risk
4. High risk

ND5 What aspect of the 2019/2020 floods caused you the greatest concern? Select up to 3

1. Damage to your property
2. Damage to the property of other family members
3. The need to evacuate
4. Risk of injury or illness to yourself
5. Risk of injury or illness to a family member
6. Economic impacts
7. Other concerns (please specify) ______

IF ND1 =3

ND6 What was the perception of risk when you became aware of COVID 19

1. No risk
2. Low risk
3. Moderate risk
4. High risk

ND7 What aspect of the COVID 19 pandemic caused you the greatest concern?

1. No vaccination available
2. The risk of illness to yourself
3. The risk of illness to a family member
4. People can carry COVID19 and not show symptoms
5. Children can be affected by COVID 19
6. The impact on your workload
7. Economic impacts
8. Other concerns (please specify)______

[PAGE BREAK]

PA1

In the past week, on how many days have you done a total of 30 minutes or more of physical activity, which was enough to raise your breathing rate? *This may include sport, exercise, and brisk walking or cycling for recreation or to get to and from places, but should not include housework or physical activity that may be part of your job.*

__________________ days

[PAGE BREAK]

D1How many serves of vegetables do you usually eat each day? One serve is half a cup cooked or 1 cup of salad vegetables.

If you do not eat vegetables, please enter 0.

| Serves | Frequency |
| --- | --- |
|  | Per day  Per week  Don’t eat vegetables |

D 2 How many serves of fruit do you usually eat per day?

If you do not eat fruit, please enter 0.

| Serves | Frequency |
| --- | --- |
|  | Per day  Per week  Don’t eat fruit |

D3 How many cups of soft drink, cordials or sports drink, such as lemonade or Gatorade do you usually drink in a day?

If you do not drink any of these, please enter 0.

| Cups | Frequency |
| --- | --- |
|  | Per day  Per week  Don’t drink these |

[PAGE BREAK]

The following questions are about tobacco smoking. This includes cigarettes, cigars and pipes. Please note that this does not include electronic cigarettes.

T1 Which of the following best describes your smoking status?

1. I smoke daily
2. I smoke occasionally
3. I don't smoke now, but I used to
4. I've tried it a few times but never smoked regularly
5. I've never smoked

T2 Which of the following best describes how often you use electronic cigarettes?

1. I've never used electronic cigarettes
2. I've tried electronic cigarettes a few times but never used them regularly
3. I don't use electronic cigarettes now, but I used to
4. I use electronic cigarettes occasionally
5. I use electronic cigarettes daily

[PAGE BREAK]

A1 How often do you usually drink alcohol?

Daily  (1)

Weekly  (6)

Monthly  (2)

Less than once per month  (3)

I don't drink alcohol  (4) [skip to next section]

Prefer not to say  (5)  [skip to A4]

If A1 = 6;

Days per week? ____

If A1 = 2;

Days per month? ____

Alcoholic drinks are measured in terms of a “standard drink”. A standard drink is usually equal to 1 middy of full-strength beer, 1 schooner of light beer, 1 small glass of wine or one pub sized nip of spirits.

If A1 = 6, 2 or 3

A2 On a day when you drink alcohol, how many standard drinks do you usually have?

____ drinks

If A1 = 1

A3 How many standard drinks per day?

____ drinks

A4 In the past four weeks have you had more than 2 standard drinks in a day?

1. Yes
2. No

A5 In the past four weeks have you had more than 4 standard drinks one occasion?

1. Yes
2. No

[PAGE BREAK]

S1 In general, how would you rate your sleep quality?

1. Poor
2. Fair
3. Good
4. Very good
5. Excellent

S2 Thinking about just the past 7 days, what time did you most often go to bed on workdays? Please answer about weekdays if you did not work last week.

S3 What about on non-work days or weekends – what time did you most often go to bed on those days?

S4 What time did you most often wake up for the day on work days or weekdays?

S5 What about on non-work days or weekends – what time did you most often wake up for the day on those days?

[PAGE BREAK]

K10 The next questions are about how you have been feeling in the past 4 weeks

|  | None of the time | A little of the time | Some of the time | Most of the time | All of the time |
| --- | --- | --- | --- | --- | --- |
| In the past 4 weeks about how often did you feel tired out for no good reason? |  |  |  |  |  |
| In the past 4 weeks, about how often did you feel nervous? |  |  |  |  |  |
| In the past 4 weeks, about how often did you feel so nervous that nothing could calm you down? |  |  |  |  |  |
| In the past 4 weeks, about how often did you feel hopeless? |  |  |  |  |  |
| In the past 4 weeks, about how often did you feel restless or fidgety? |  |  |  |  |  |
| In the past 4 weeks, about how often did you feel so restless you could not sit still? |  |  |  |  |  |
| In the past 4 weeks, about how often did you feel depressed? |  |  |  |  |  |
| In the past 4 weeks, about how often did you feel that everything was an effort? |  |  |  |  |  |
| In the past 4 weeks, about how often did you feel so sad that nothing could cheer you up? |  |  |  |  |  |
| In the past 4 weeks, about how often did you feel worthless? |  |  |  |  |  |

If at any time you are experiencing personal distress and would like crisis support you can call Lifeline on 13 11 14 or Beyond Blue on 1300 22 4636

[PAGE BREAK]

TSWQ1 Below are some questions about your experiences as a teacher.

Read each sentence and choose the ​one​ response that best describes you.

|  | Almost Never | Sometimes | Often | Almost Always |
| --- | --- | --- | --- | --- |
| 1. I feel like I belong at this school. |  |  |  |  |
| 2. I am a successful teacher. |  |  |  |  |
| 3. I can really be myself at this school. |  |  |  |  |
| 4. I am good at helping students learning new things. |  |  |  |  |
| 5. I feel like people at this school care about me. |  |  |  |  |
| 6. I have accomplished a lot as a teacher. |  |  |  |  |
| 7. I am treated with respect at this school. |  |  |  |  |
| 8. I feel like my teaching is effective and helpful. |  |  |  |  |

[PAGE BREAK]

TBS1 The following questions ask about your experience as a teacher.

|  | Strongly disagree (1) | Moderately disagree (2) | Slightly disagree (3) | Slightly agree (4) | Moderately agree (5) | Strongly agree (6) |
| --- | --- | --- | --- | --- | --- | --- |
| I look forward to teaching in the future (1) |  |  |  |  |  |  |
| I feel depressed because of my teaching experiences (2) |  |  |  |  |  |  |
| I get adequate praise from my supervisors for a job well done (3) |  |  |  |  |  |  |
| The teaching day seems to drag on and on (4) |  |  |  |  |  |  |
| I am glad that I selected teaching as a career (5) |  |  |  |  |  |  |
| The students act like a bunch of animals (6) |  |  |  |  |  |  |
| My physical illnesses may be related to the stress in this job (7) |  |  |  |  |  |  |
| I feel that the administrators are willing to help me with classroom problems, should they arise (8) |  |  |  |  |  |  |
| I find it difficult to calm down after a day of teaching (9) |  |  |  |  |  |  |
| Teaching is more fulfilling than I had expected (10) |  |  |  |  |  |  |
| I believe that my efforts in the classroom are unappreciated by the administrators (11) |  |  |  |  |  |  |
| If I had it to do all over again, I would not become a school teacher (12) |  |  |  |  |  |  |
| I feel that I could do a much better job of teaching if only the problems confronting me were not so great (13) |  |  |  |  |  |  |
| The stresses in this job are more than I can bear (14) |  |  |  |  |  |  |
| My supervisors give me more criticism than praise (15) |  |  |  |  |  |  |
| Most of my students are decent people (16) |  |  |  |  |  |  |
| Most students come to school ready to learn (17) |  |  |  |  |  |  |
| I feel that the administrators will not help me with classroom difficulties (18) |  |  |  |  |  |  |
| I look forward to each teaching day (19) |  |  |  |  |  |  |
| The administration blames me for classroom problems (20) |  |  |  |  |  |  |
| Students come to school with bad attitudes (21) |  |  |  |  |  |  |

[PAGE BREAK]

The following questions ask about your use of health-related technology.

HT 1 Do you use any health or wellbeing apps?

Sports and fitness activity tracking  (1)

Diet and nutrition  (2)

Weight loss coaching  (3)

Pharmacy  (4)

Sleep cycle analysis  (5)

Stress reduction and relaxation  (6)

Meditation  (7)

Medical advice and patient community  (8)

Menstrual period tracking  (9)

Pregnancy  (10)

Hospital selection and appointment management  (11)

Smoking cessation  (16)

Reduce alcohol consumption  (17)

Other (please specify)  (12) ________________________________________________

I don't use any health or wellbeing apps  (13)

[PAGE BREAK]

If HT1 = 1,2,3,4,5,6,7,8,9,10,11,16 or 17;

HT2 How frequently do you use these apps?

| App category | Frequency of use | Length of use |
| --- | --- | --- |
|  |  |  |
| Sports and fitness activity tracking (x1) | ▼ Daily  Weekly  Monthly  Yearly | ▼ Less than 1 week  1- 2 weeks  3- 4 weeks   1. 2 months   3 – 6 months  6months – 1 year  1+years |
|  |  |  |

[PAGE BREAK]

HT3 Have you ever used a physical activity tracker (e.g. Fitbit, Garmin, Apple watch)?

1. Yes
2. No

IF HT3= 1

HT4 Do you currently use a physical activity tracker (e.g. Fitbit, Garmin, Apple watch)?

1. Yes
2. No

HT5 For how long have you used a physical activity tracker?

- Up to 3 months
- More than 3, up to 6 months
- More than 6 months, up to 1 year
- More than 1 year, up to 2 years
- Don’t know

HT6 What do you predominantly use your physical activity tracker for? (select up to 3)

- Measuring the number of steps you take in a day
- The distance or speed you travel while running or cycling
- Measuring your heart rate
- Measuring calories burned
- Sleep tracking
- Swimming training
- Movement reminders
- Workout coaching
- Smartphone integration (providing notifications of call/texts, etc)
- Other. Please specify

HT7 Were you already active in sport and other physical activity before you started using a physical activity tracker?

1. Yes
2. No
3. Don’t know

HT8 In general, would you say that the physical activity tracker has a major influence, a minor influence or no influence at all on the physical activity that you do?

- Major influence
- Minor influence
- No influence at all
- Don’t know

HT9 Do you use your physical activity tracker to compare your activity levels to other people?

- Yes
- No

IF HT4=no

HT10You said that you used to use a physical activity tracker. What was the main reason you stopped using it?

- Wasn’t comfortable to wear
- Too much hassle
- Wasn’t having a sufficiently positive impact on my activity levels
- Friends/ contacts stopped using it
- I got bored with it
- It broke and I didn’t replace it
- It was inaccurate
- Other. Please specify
- Don’t know

IF HT4=no

HT5 For how long did you use your physical activity tracker before you stopped using it?

- Up to 3 months
- More than 3, up to 6 months
- More than 6 months, up to 1 year
- More than 1 year, up to 2 years
- Don’t know

[PAGE BREAK]

The following questions ask about your intention to stay in the teaching profession.

W1 How many more years do you think you will be in the teaching profession?

1. 1 – 2 years
2. 3 – 5 years
3. 6 – 9 years
4. 10+ years
5. Unsure

W2 Do you think you will stop working in schools (in any capacity) permanently before you retire?

1. Yes
2. No [Next section]
3. Unsure

W3 if you were ever to leave the teaching profession, what would be some of the reasons for that? *Please select all that apply*

1. I never intended teaching to be a long-term career
2. I have found that I am not suited to working in schools
3. I am not enjoying working in schools
4. Unsatisfactory relationships with other staff
5. To seek employment elsewhere in education, but not directly in schools
6. To seek employment outside of education
7. The workload is too heavy
8. Insufficient support staff
9. Class sizes too large
10. I'm facing challenges with student behaviour management
11. Insufficient professional recognition within the school
12. The poor public image of the profession
13. Changes imposed on schools from outside (e.g. from government)
14. Dissatisfaction with performance appraisal processes
15. The demands of professional regulation (e.g. professional learning, practice, etc.) are too heavy
16. Family Reasons / Parental leave
17. I am finding it too stressful / impacting my well-being or mental health
18. To achieve a better work / life balance
19. Insufficient pay
20. Other

[PAGE BREAK]

D1 What is your sex?

1. Male
2. Female
3. Other
4. Prefer not to say

D2 How old are you?

1. 20-24
2. 25-29
3. 30-34
4. 35-39
5. 40-44
6. 45-49
7. 50-55
8. 56-59
9. 60-64
10. 65 years or older
11. I prefer not to answer

D3 Whendid you complete your initial education qualification?

- 0-2 years ago
- 3-5 years ago
- 6-10 years ago
- 10-15 years ago
- 16-20 years ago
- Over 20 years ago

D5 What is the highest level of education that you have completed?

1. No formal qualifications
2. School certificate or equivalent
3. Higher school certificate or equivalent
4. TAFE qualifications
5. Diploma or advanced diploma
6. Bachelor Degree
7. Graduate certificate or Graduate diploma
8. Masters Degree
9. PhD

[PAGE BREAK]

D6 Including yourself, how many adults (18 years and over) and children (under 18 years ) are in your household?

______ adults

______ children

[PAGE BREAK]

D7a What is your height?

- in centimeters  (1)
- in feet and inches  (2)

IF D7a = 1

D7b  Please enter your height in cm (e.g. if 1.75m, enter 175

________________________________________________________________

IF D7a = 2

D7c Please enter your height in feet (e.g. 5'6 enter 5 here)

________________________________________________________________

D7 c Please enter your height in inches  (e.g. 5'6 enter 6 here)

________________________________________________________________

[PAGE BREAK]

D8a What is your weight?

- in kilograms  (1)
- in stones and pounds  (2)

IF D8a = 1

D8b Please enter your weight in kilograms (kg)

________________________________________________________________

IF D8b = 2

D8c Please enter your weight in stones (e.g. if 11 stone 5 pound, enter 11) 
______________________

D8c Please enter your weight in pounds (e.g. if 11 stone 5 pound, enter 5)

________________________________________________________________

[PAGE BREAK]

This survey remains confidential and anonymous.

C1 Thank you for our time completing the NSW Teachers’ Health Survey! As part of our appreciation for your time we would like to enter you into a small competition to win 1 of 20 $100 VISA gift vouchers. We will be contacting the winners by email in December 2020. Would you like to enter the prize draw to win 1 of 20 $100 VISA gift vouchers?

1. Yes – I would like to enter the prize draw
2. No – I do not want to enter the prize draw

C2 Would you like to be provided with a summary of the general results from this study on teachers' health in NSW?

1. Yes – please send me a summary of results
2. No – I don’t want to be sent results of this study

C3 Are you interested in participating in future studies on teachers’ health?

1. Yes – I please contact me regarding future studies on teachers’ health
2. No – I don’t want to be contacted further

IF C1 =1 or C2 =1 or C3= 1

C4 Please provide us with your contact details.

First name:

Last name:

Email address:
