# Supplementary material for: A cross-sectional study of Australian teachers’ health: are work-related factors associated with lifestyle behaviours?
Source: Health Promot Int. 2024 Jan 10;39(1):daad192. doi: 10.1093/heapro/daad192 (PMC10781439; doi:10.1093/heapro/daad192)
Supplement: daad192_suppl_Supplementary_Figures_S2 [file daad192_suppl_supplementary_figures_s2.docx]

Supplementary Material S2. Sensitivity analysis

Supplementary Table 1: Odds of being classified as ‘healthy’ on lifestyle index (including alcohol consumption) based on various work-related factors after adjusting for sex, age, BMI, geography and having children. Cut point of 4+ healthy lifestyle behaviours were used to classify teachers as healthy. *=p<0.05, **=p<0.01, ***= p<0.001

| **Work-related factors** | **Classified as ‘healthy’ on lifestyle index** | |
| --- | --- | --- |
|  | OR(95% CI) | p |
| **Hours worked/week**  <40hrs (ref)  40-49hrs  50-59hrs  60+hrs | 1.07 0.64 1.79  0.69 0.43 1.11  0.62 0.37 1.04 | <0.05 |
| **Teaching experience**  < 5 years (ref)  6-15 years  16+years | 1.22 0.75 1.98  1.29 0.72 2.29 | 0.67 |
| **Teaching load**  Full time (ref)  Part time | 1.56 1.07 2.26 | <0.05 |
| **Contract type**  Permanent (ref)  Fixed term/casual | 1.09 0.78 1.52 | 0.63 |
| **School Sector**  Independent (ref)  Government  Catholic | 0.73 0.48 1.10  0.76 0.47 1.25 | 0.31 |
| **Teacher role**  Teacher (ref)  Leadership position | 0.87 0.61 1.22 | 0.42 |
| **Teacher type**  Primary  Secondary | 1.19 0.86 1.64 | 0.28 |
| **Perceived risk of COVID-19**  No/low risk (ref)  Moderate risk  High risk | 1.14 0.80 1.62  0.93 0.58 1.48 | 0.61 |
